# Defects in GnRH Neuron Migration/Development and Hypothalamic-Pituitary Signaling Impact Clinical Variability of Kallmann Syndrome

**DOI:** 10.3390/genes12060868

**Published:** 2021-06-05

**Authors:** Małgorzata Kałużna, Bartłomiej Budny, Michał Rabijewski, Jarosław Kałużny, Agnieszka Dubiel, Małgorzata Trofimiuk-Müldner, Elżbieta Wrotkowska, Alicja Hubalewska-Dydejczyk, Marek Ruchała, Katarzyna Ziemnicka

**Affiliations:** 1 Department of Endocrinology Metabolism and Internal Medicine, Poznan University of Medical Sciences, 61-701 Poznan, Poland; mkaluzna@ump.edu.pl (M.K.); elawrot@vp.pl (E.W.); mruchala@ump.edu.pl (M.R.); kaziem@ump.edu.pl (K.Z.); 2 Department of Reproductive Health, Centre of Postgraduate Medical Education, 01-813 Warsaw, Poland; mirab@cmkp.edu.pl; 3 Department of Otolaryngology Head and Neck Surgery, Poznan University of Medical Sciences, 61-701 Poznan, Poland; jarek.kaluzny@gmail.com; 4 Chair and Department of Endocrinology, Jagiellonian University Medical College Krakow, 30-688 Krakow, Poland; ociepka.agnieszka@gmail.com (A.D.); malgorzata.trofimiuk@uj.edu.pl (M.T.-M.); alahub@cm-uj.krakow.pl (A.H.-D.)

**Keywords:** Kallmann syndrome (KS), isolated hypogonadotropic hypogonadism (IHH), next-generation sequencing (NGS), combined pituitary hormone deficiency (CPHD), GnRH neuron, anosmia, hyposmia

## Abstract

Kallmann syndrome (KS) is a combination of isolated hypogonadotropic hypogonadism (IHH) with olfactory dysfunction, representing a heterogeneous disorder with a broad phenotypic spectrum. The genetic background of KS has not yet been fully established. This study was conducted on 46 Polish KS subjects (41 males, 5 females; average age: 29 years old). The studied KS patients were screened for defects in a 38-gene panel with next-generation sequencing (NGS) technology. The analysis revealed 27 pathogenic and likely pathogenic (P/LP) variants, and 21 variants of uncertain significance (VUS). The P/LP variants were detected in 20 patients (43.5%). The prevalence of oligogenic P/LP defects in selected genes among KS patients was 26% (12/46), whereas the co-occurrence of other variants was detected in 43% (20 probands). The examined KS patients showed substantial genotypic and phenotypic variability. A marked difference in non-reproductive phenotypes, involving defects in genes responsible for GnRH neuron development/migration and genes contributing to pituitary development and signaling, was observed. A comprehensive gene panel for IHH testing enabled the detection of clinically relevant variants in the majority of KS patients, which makes targeted NGS an effective molecular tool. The significance of oligogenicity and the high incidence of alterations in selected genes should be further elucidated.

## 1. Introduction

Kallmann syndrome (KS) is a rare hormonal disorder of sexual maturation and fertility. Smell disturbances (hyposmia or anosmia) are an indispensable part of KS. Patients with KS constitute approximately 60% of subjects with isolated hypogonadotropic hypogonadism (IHH) [1]. Gonadotropin-releasing hormone (GnRH) neuron development and migration disturbances or dysfunction represent the core background of the disease. The origin of GnRH neurons is a nasal placode—an embryonal region that invaginates to establish the olfactory epithelium and the vomeronasal organ, giving rise to the formation of olfactory sensory neurons and olfactory ensheathing cells [2]. During embryogenesis, GnRH neurons from the vomeronasal organ travel along the axons surrounded by olfactory ensheathing cells to the forebrain—a final destination for governing hormone signaling through the hypothalamic–pituitary–gonadal (HPG) axis and contributing to proper sexual advancement [3]. The common origin of GnRH and olfactory neurons, and disturbances in the development of olfactory placodes, deliver reasonable explanation for the associated occurrence of impaired sense of smell (anosmia, hyposmia) in KS patients comparing to other IHH forms. KS might be also accompanied by diverse congenital defects, including midline cranial anomalies, dental agenesis, renal defects, and limb malformations [4,5,6]; its most complex form is known as CHARGE syndrome—a congenital disorder associated with coloboma, heart malformation, choanal atresia, retardation of growth and/or development, genital anomalies, and ear anomalies/deafness. Because of the overlapping clinical manifestation, IHH and olfactory defects are today considered to be part of CHARGE syndrome [7,8]. This phenotype suggests the involvement of broader complex embryonic disturbances than those related to the HPG axis, and various cellular processes [9]. So far, more than 50 genes involved in the development of the hypothalamus and pituitary gland, formation and migration of GnRH neurons, or the regulation of GnRH and gonadotropin secretion have been described. Due to the contribution of genes located on the X chromosome, hypogonadotropic hypogonadism is five times more common in males than in females. About 1 in 30,000 men and 1 in 125,000 women are affected by KS [10,11,12]. The inheritance patterns for KS encompass X-linked recessive, autosomal dominant, and autosomal recessive transmission. Digenic and oligogenic defects are increasingly reported [13,14], constituting a major explanation for the clinical variability of the disorder. Moreover, the genetic background for barely half of IHH cases is established, and new genetic targets are still being sought for IHH patients [14,15,16]. KS/IHH is a genetically and phenotypically heterogeneous disease whose incidence, as well as the phenotypic and genetic spectrum of KS/IHH for the Polish population, is limited [17,18,19,20,21,22,23].

### Objective

The current study aimed to investigate the genetic basis of KS in adult Polish patients. The study also sought to report the ratios of pathogenic or likely pathogenic (P/LP) alterations, variants of uncertain significance (VUS), and benign or likely benign (*B/LB*) alterations, according to the American College of Medical Genetics and Genomics (ACMG)’s classification. The objective of the study was to establish genotype–phenotype correlations, as well as to identify variant recurrence among KS patients in Poland.

## 2. Materials and Methods

### 2.1. Patients

A group of 46 well-characterized patients (41 males, 5 females; average age: 29; min–max: 18–57 years) with KS were included in the study and subjected to genomic screening. Phenotypic information was based on clinical interview and physical examination, including anthropometric measurements. The history of unilateral or bilateral cryptorchidism was collected. The patients were subjected to an andrological assessment (physical examination and ultrasound examination of the testicles) or a gynecological examination. In each case, an abdominal ultrasound examination was conducted in order to detect additional congenital defects. The reversibility of KS in male subjects was assessed as the capability to achieve a normal adult testosterone serum concentration after six months of hormone replacement therapy withdrawal [24]. Pubertal status was specified as lack of puberty, incomplete puberty, or complete puberty [25].

To assess pituitary function, the measurements of basic and, if necessary, stimulated concentrations of pituitary hormones—luteinizing and follicle-stimulating hormones (LH, FSH), growth hormone (GH), adrenocorticotropic hormone (ACTH), thyrotropin (TSH)s and prolactin (PRL)—were carried out. Measurements of testosterone, estradiol, morning cortisol, dehydroepiandrosterone sulfate (DHEA-S), insulin-like growth factor 1 (IGF-1), free triiodothyronine (fT3), and free thyroxin (fT4) in fasting blood serum were also taken. Serum cortisol level was also measured at 6:00 p.m. Hormonal measurements were performed with a Cobas 6000 (Roche Diagnostics, Basle, Switzerland) using dedicated electrochemiluminescence sandwich immunoassay (ECLIA) kits provided by the manufacturer. The following calculation determined the free testosterone index (FTI): (*FTI*) = 100 × (total testosterone/SHBG). A test with 100 μg of intravenous gonadoliberin (LHRH, Ferring) was conducted in each patient. Hypogonadotropic hypogonadism was diagnosed in all patients according to the European Consensus Statement (2015), and was based on the presence of low serum levels of testosterone in male individuals (3.5 nmol/L) or low estradiol levels (<20 pg/mL) in females, associated with serum levels of gonadotropins of less than 5 international units/L, without reaction to GnRH [25].

No other associated pituitary endocrinopathy was identified in the studied subjects. The body weight and height of the patients were measured using a height-measuring stand with a weight and height scale machine to obtain the anthropometric data. BMI was calculated according to the Quetelet formula: BMI (kg/m^2^) = weight (kg)/height (m^2^).

All patients underwent ENT assessment and psychophysical testing of the olfactory function. Olfactometry was performed according to Elsberg and Levy’s method, modified by Pruszewicz [26,27]. Elsberg and Levy’s method, enabling detection and recognition thresholds for the four odorants, is the most widely used and available smell-testing method in Poland. All patients were checked for presentation of any features of the CHARGE spectrum.

### 2.2. Genetic Studies

Molecular studies were conducted using next-generation sequencing (NGS) technology with the Ion Torrent Personal Genome Machine system (Ion PGM ™, Thermo Fisher Scientific, Waltham, MA, USA). The targeted panel of 38 genes associated with IHH and combined pituitary hormone deficiency (CPHD) encompassed: *ADAM7*, *ANOS1*, *BMP2*, *BMP4*, *CHD7*, *FGF8*, *FGF17*, *FGFR1*, *GLI2*, *GNRH1*, *GNRHR*, *HESX1*, *HS6ST1*, *IGSF10*, *KISS1*, *KISS1R*, *LEP*, *LEPR*, *LHB*, *LHX3*, *LHX4*, *LRRIQ3*, *NSMF*, *NR0B1*, *OTX1*, *OTX2*, *PCSK1*, *PITX1*, *PITX2*, *PROK2*, *PROKR2*, *PROP1*, *POU1F1*, *SEMA3A*, *SOX3*, *TAC3*, *TACR3*, and *WDR11*.

The sequences were mapped to the human genome (GRCh37/hg19) using Torrent Suite ™ software (version 4.0.2, Thermo Fisher Scientific, Waltham, MA, USA), and the variant positions were then translated to (GRCh38/hg38) when needed. Each detected variant was assigned according to HGVS nomenclature with the use of canonical transcripts (according to Ensembl). The analysis of the obtained sequences was made using the following algorithms: PhenIX (http://compbio.charite.de/PhenIX/), Mutation Taster 2 (http://www.mutationtaster.org/), and Variant Effect Predictor (VEP) in Ensembl (19), assuming prediction from FATHMM, CAD, PolyPhen2, SIFT, and MaxEntScan. For pathogenicity evaluation, the clinical databases HGMD (http://www.hgmd.cf.ac.uk) and ClinVar (https://www.ncbi.nlm.nih.gov/clinvar/) were also checked. The GnomAD database (https://gnomad.broadinstitute.org/) was explored to establish the reported presence and frequency of variants. The minor allele frequency (MAF) of < 1% in GnomAD was assumed as the primary criterion for pathogenicity examination. Alterations were classified according to updated guidelines reported by the ACMG—pathogenic and/likely pathogenic (P/LP), variant of unknown clinical significance (VUS), and benign and/likely benign (B/LB)—assuming impact on protein functionality, evolutionary conservativeness, and frequency. Varsome [28] and InterVar [29] were used for correctness of position estimation and HGVS nomenclature. The detected mutations were confirmed using conventional capillary sequencing. Selected mutations were also subjected to in silico analysis in order to evaluate the effect of the change on protein architecture, considering its functionality and potential involvement in the manifestation of an abnormal phenotype using PHYRE2 (http://www.sbg.bio.ic.ac.uk) and Chimera v1.7 [30].

## 3. Results

### 3.1. Characteristics of the Group

The analyzed KS group had a high male predominance (M:F ratio of 8:1). Four familial cases of KS were included in the study. A severe reproductive phenotype was observed in the majority of patients. The prevalence of a total lack of puberty reached 78%. Incomplete puberty was reported in 9% of subjects. In contrast, complete puberty was seen in 13% of patients. Hyposmia was present in 28% (13/46) of patients, and 58% (27/46) of patients were anosmic, according to the psychophysical smell test. The 36% (15/41) of male patients had diagnosed cryptorchidism—unilateral in six cases and bilateral in nine patients. The prevalence of the additional congenital defects, apart from dysosmia, was 45%. The most common abnormalities included renal defects (renal agenesis, horseshoe kidney, double pyelum; five cases), limb malformations (syndactyly, clinodactyly, brachydactyly; five cases), and mirror movements (three cases). The incidence of reversible IHH forms was noted for 6% of cases (three cases). Detailed clinical data are presented in Table 1 and Table 2.

### 3.2. Genetic Results

**Figure 1:**
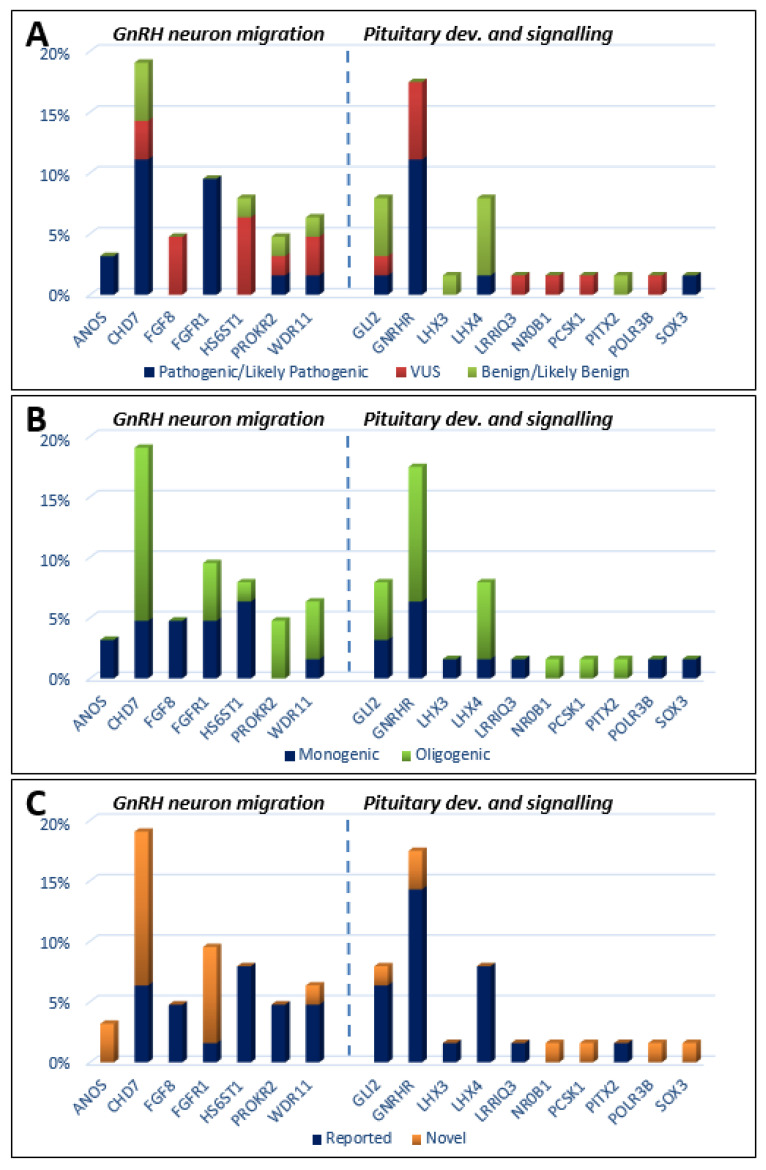
Histograms showing the prevalence of mutations among KS patients, which are classified by three metrics: (**A**) mutation severity (ACMG criteria: pathogenic/likely pathogenic, VUS variant of unknown significance, benign/likely benign); (**B**) oligogenicity; (**C**) novelty.

Genetic defects were classified as those related to GnRH neuron development and migration (Table 1), and those related to pituitary development and signaling (Table 2). Defects in 18 genes were detected for 46 KS patients. The 50 identified variants were re-graded as clinically relevant, of which 11 were classified as pathogenic (P), 12 as likely pathogenic (LP), 16 as VUS, and 11 as benign or likely benign (B/LB) (Figure 1A). Heterozygous P/LP alterations were found in the *CHD7* (*n*=7; 15.2% of all KS subjects, all novel and not reported); *FGFR1* (*n* = 6; 13%, 5 novel), *GNRHR* (*n* = 4; 9%, 2 novel), *GNRH1* (*n* = 1; 2%), *WDR11* (*n* = 1; 2%), *PROKR2* (*n* = 1; 2%), *GLI2* (*n* = 1; 2%), and *LHX4* (*n* = 1; 2%) genes. Hemizygous P/LP variants were detected in the *ANOS1* (*n* = 1, familial case (two patients); 2%) and *SOX3* (*n* = 1; 2%) genes (Table 1 and Table 2). For six IHH patients (two with hyposmia and four with anosmia), no causative defects were identified at all. Monogenic variants with clinically significant P/LP alteration were seen in eight KS patients and in another seven subjects presenting oligogenicity (Figure 1B). In total, 15 novel pathogenic variants, not previously reported for KS, were detected in 19 patients affecting 7 genes (*ANOS1*, *CHD7*, *FGFR1*, *GLI2*, *GNRHR*, *SOX3*, and *WDR11*) (Figure 1C).

**Figure 2:**
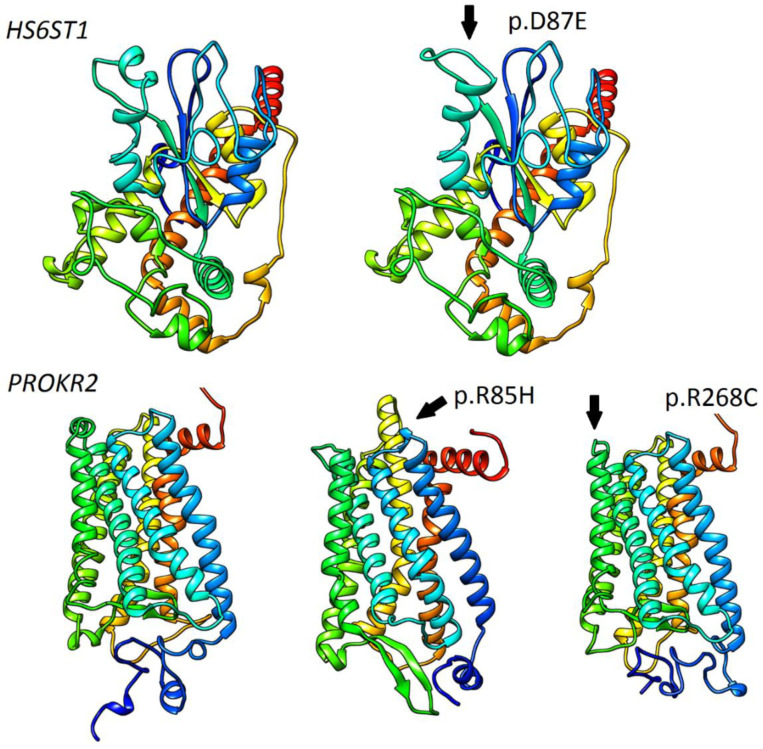
Modelling and prediction of conformational changes of selected *HS6ST1* and *PROKR2* variants. Predicted conformational changes surrounding variant positions are highlighted and indicated with arrows. Ribbon models of reference *HS6ST1* and *PROKR2* are shown on the left side.

Concerning variant recurrence, the heterozygous mutation in *FGFR1* p.R285W (likely pathogenic; CM063987 HGMD), coexisting with another heterozygous variant *CHD7* p.N1030H (pathogenic; novel), was recurring in two unrelated patients, both with reversal of hypogonadism. A heterozygous variant in *LHX4* p.D128= (benign; MAF 0.006, rs141139762) was detected in three cases (four patients, including two affected brothers). In summary, the applied approach identified 18 P/LP variants in genes responsible for the development and migration of GnRH neurons (Table 1) and 9 P/LP variants in genes contributing to pituitary formation and pituitary/hypothalamic signaling (Table 2). P/LP variants in pituitary genes were identified for two of the examined KS patients: an oligogenic defect in *LHX4* (p.G305W)/*CHD7* (p.K850Q), and proband 34 with a p.R155Afs*26 defect in *SOX3* (Table 1 and Table 2). Detailed information referring to identified variants, comprising their precise genomic coordinates and functional prediction, is included in the Table S1. In order to further explore the involvement of *HS6ST1* (p.D87E, p.R249S) and *PROKR2* (p. R85H, p.R268C) variants, in silico modelling was performed. Except for *HS6ST1*p.R249S (low confidence of prediction), changes in protein architecture were noted for all mentioned variants (Figure 2).

### 3.3. Genotype–Phenotype Correlations

Associated non-reproductive malformations were much more common in patients with defects in the genes governing GnRH neuron development and migration than in patients with defects associated with pituitary and signaling cascades (Table 1 and Table 2). Limb malformations were recorded only in the P/LP-variants-positive group, with defects in *CHD7*, *FGFR1*, *HS6ST1*, and *LHX4*. The majority of patients with digital bone abnormalities presented heterozygous mutation in *CHD7* (80% of cases). Similarly, renal abnormalities were detected only in the P-variant-positive group (six patients with mutations in *ANOS1*, *CHD7*, *FGFR1*, and *GNRHR*). Synkinesis was seen in three cases with mutated *ANOS1* (family case), *FGF8*, and digenic defects in *GNRHR* and *WDR11*. Dental agenesis was detected in the male patients with digenic defects in *CHD7* and *LHX4* (proband 8). Additional CHARGE features were present in two analyzed KS patients with P/LP variants in *CHD7*: proband 13 (choanal atresia), and proband 21 (ear anomaly).

### 3.4. Male Reversibility

In a long-term clinical follow-up, three unrelated subjects with digenic defects (proband 9: *CHD7* p.N1030H and *FGFR1* p.R285W; proband 10: *CHD7* p.N1030H and *FGFR1* p.R285W; and proband 25: *GNRHR* p.R139H, *GNRHR* p.N10_Q11delinsKK, and *CHD7* p.K683_T684insAK) displayed a reversal of hypogonadism. Two of them had hyposmia. Only one of them had an additional congenital deformity (sandal gap) and a KS family history.

### 3.5. Females

In the current cohort, P/LP variants were found in two out of five female patients, affecting the *GNRH1* and *GNRHR* genes. The two anosmic KS females with a history of primary amenorrhea harbored P/LP variants (in the first case in the *GNRH1* gene (with no other accompanying congenital defect), and in the second case in the *GNRHR* gene), presenting incomplete rotation of the right kidney. (Table 2)

## 4. Discussion

### 4.1. Effectiveness of Panel-Based NGS

The study was conducted on 46 adult patients with Kallmann syndrome. The panel-based NGS approach used in this study appears to be an efficient and cost-effective method. A panel of 38 genes enabled the detection of causative variants (P/LP) in 43.5% of KS patients. The frequency of new P/LP variants was high: 65% of all detected P/LP variants, and in 37% of patients (17/46). VUS alterations were detected in 37% of studied subjects. The failure to find P/LP variants In 56% of enrolled subjects could explain mutations outside the coding regions or mutations in other IHH candidate genes [16].

The overall effectiveness of the application of NGS with a panel of 38 IHH/CPHD genes in the current study was similar to or even higher than the efficacy of the panel-based NGS approach in previous studies. Targeted NGS was also applied by Zhou et al. in 148 Chinese male IHH patients. Zhou et al. used a panel of 83 genes (31 known IHH genes and 52 candidate genes) and identified P/LP variants in 14.9% of patients, in 16 causal genes and 36 candidate genes. The results of targeted NGS of 261 candidate genes in 48 subjects with KS/nIHH with no mutations in any known KS/nIHH genes were described by Quyanor et al., who detected two new LP variants in *FGFR1*, and proposed 18 new candidate genes in KS/nIHH [31]. Butz et al. employed a panel of 41 genes to analyze the genetic backgrounds of 38 patients with hypogonadotropic hypogonadism, and found genetic defects in 8 patients (21%) [32]. Gach et al. identified known or novel potentially deleterious variants in 23 out of 47 (49%) unrelated IHH patients (31 men, 16 women; 26 nIHH and 23 KS) tested with NGS using a 51-gene panel. In the next step of the Gach et al. study, a WES analysis and molecular diagnosis were completed in 19/47 CHH cases. Monogenic mutations were found in 13 subjects in *ANOS1*, *FGFR1*, *GNRHR*, *CHD7*, *SOX10*, and *PROKR2*, whereas in 6 patients oligogenic variants were identified in *SPRY/SEMA3A*, *CCDC141/POLR3B*, *SRA1/SEMA7A*, *CHD7/SEMA71*, *NSMF/SPRY4/PROKR2*, and *TACR3/LHB* [22]. Cangiano et al. examined 160 males with classic or milder ao-IHH—both nIHH and KS—using NGS and a panel of 28 genes. In that study, rare gene variants were detected in 55% of patients (although it was 32% considering only the 13 first-discovered IHH genes (*ANOS1*, *FGFR1*, *PROKR2*, *PROK2*, *GNRHR*, *GNRH1*, *GNRH2*, *KISS1*, *KISS1R*, *TAC3*, *TACR3*, *HS6ST1*, *FGF8*)) [33]. The effectiveness of the panel used in the current study was so high due to the careful selection of only patients with KS, as well as the designed NSG gene panel, which covered the most commonly mutated genes.

### 4.2. Defects in Genes Responsible for GnRH Neuron Migration and Development

Previously reported and new genetic factors involved in the etiopathogenesis of KS have been identified in the studied patients. P/LP variants in genes involved in neuron migration (*ANOS1*, *CHD7*, *FGFR1*, and *WDR11*) were found predominantly in the current study. The most common P/LP variants in Polish probands were found in the *CHD7* and *FGFR1* genes. Furthermore, the most severe reproductive phenotype was seen in patients with alterations in those genes, particularly when accompanying variants in other IHH genes were present. The *CHD7-* and *FGFR1-*positive patient group was significantly enriched by additional non-reproductive congenital malformations. The 15% prevalence of P/LP variants of *CHD7* in Polish KS patients is higher than the reported incidence of 4–10% in other IHH studies [22,34,35]. In addition, the incidence of mutations in *FGFR1* appears to be higher than in previous reports—13% vs. 8.5% [22]. The rate of mutations in *GNRHR*, *GNRH1*, and *PROKR2* was similar to that reported in the literature. In contrast, mutations in *WDR11*, *HS6ST1*, and *FGF8* were recognized in the current study slightly more often than in previous literature [34,36], although one must consider the number of KS patients analyzed in the current study.

Patients enrolled in the current study were characterized by notable genetic and phenotypic variability. Even the presence of the same P/LP variant appears to result in variable expressivity (e.g., patients 1 and 2 (family case), patients 9 and 10 (unrelated subjects)). The genetic heterogeneity of gene defects in *CHD7*, *FGFR1*, and *GNRHR* was observed in the current study. Genotypic and phenotypic diversity of KS is broad, and varies among different populations, presenting a significant diagnostic challenge. The genetic heterogeneity of KS, especially within respective pedigrees, has been noted by multiple authors [16,37,38].

No recurrent founder mutation originating from Poland was found. The p.R285W defect in *FGFR1* was co-occurring with p.N1030H in the *CHD7* gene as a digenic case for two sporadic, unrelated patients. It is worth noting that 15 new P/LP variants were detected in known IHH genes, which emphasizes the importance of the comprehensive elucidation of known genes in various populations. An interesting case of co-occurrence of two variants in *HS6ST1* (p.D87E, p.R249S) was detected. Because variants were found in two unrelated sporadic patients, we think that this was more likely a monoallelic case (*cis* form) than compound heterozygosity (*trans*), but the DNA of the patients’ parents was unavailable, and so we could not check it. Despite the phenotypical manifestation, the lack of other relevant abnormal variants and protein modeling data for p.D87E seems to suggest clinical relevancy. Causativeness for two oligogenic variants in *PROKR2* (p.R85H, p.R268C) seems to confirm previous clinical and functional reports [39,40](Figure 2).

### 4.3. Defects in Genes Responsible for Pituitary and Hypothalamic Development and Signaling

A shared genetic background for CPHD, holoprosencephaly, and IHH/KS was proposed by Raivo et al. and Vaaralahti et al. [41,42]. Rare variants of genes implicated in the etiology of KS/IHH—*ANOS1*, *FGFR1*, *FGF8*, and *PRORK2*—were also found in patients with CPHD [41,43,44]. Therefore, the known and candidate genes responsible for pituitary gland development and CPHD were included in the gene panel used in the study (listed in Methods). Two heterozygous missense changes in the *SOX3* and *GLI2* genes, associated with holoprosencephaly, were found in KS patients—the first previously unreported in any subject without a mutation in other examined known KS/IHH genes, and the second in patients with a deleterious variant in *PROKR2*: p.R85H (c.254G>A, Figure 1). In all patients, other pituitary hormone deficiencies, except for hypogonadism, were excluded.

In eight patients from the current study (17.4%), P/LP variants were detected in *GNRH1*, *GHRHR1*, *GLI2*, *LHX4*, and *SOX3*. Particularly interesting were pathogenic variants in *SOX3:* p.R155Afs*26 (patient with hyposmia and ao-IHH, proband 34) and combined likely pathogenic variants in *LHX4*: pG305W/*CHD7*: p.K850Q in an anosmic patient with absent puberty (proband 8) (Table 1 and Table 2). Novel LP variants in the transcription factor *SOX3* (*SOX3* p.A234_240del), implicated in the etiology of septo–optic dysplasia (SOD), was observed in an nIHH patient by Kim et al. [45]. Loss-of-function mutations in another transcription factor of the SOX family, *SOX10*, were linked to KS associated with deafness [46,47,48,49]. Interestingly, heterozygous benign variants in *LHX4*: p.D128 = were recurring in four patients, of which three turned out to be oligogenic (proband 11, pathogenic variant in CHD7: p.N1030 and benign in *HS6ST1:* p.K67*; proband 13, pathogenic change in CHD7: p.D2838Tfs*51; and proband 39, benign *CHD7* variant p.S103T). Patients with defects in genes responsible for CPHD need careful follow-up and regular evaluation for hypofunction in other pituitary axes.

Two novel P/LP variants in the *GNRHR* gene were also found (p.C114* and p.N10_Q11delinsKK), both in compound heterozygosity patterns with reported *GNRHR* defects (probands 24 and 25).

The VUS variant was found in the *LRRIQ3* gene (Leucine-Rich Repeats and IQ Motif-Containing 3; LRRC44), a candidate gene for KS/IHH; its expression has been proven in the pituitary gland and the testicles, and its product is probably an intracellular protein [50]. *LRRIQ3* is linked with neurodevelopmental disorders and delayed puberty [51,52]. In the current study, the splice-site mutation in *LRRIQ3* was found in a 24-year-old patient with severe reproductive phenotype and hyposmia. The possible impact of *LRRIQ3* mutations on the development of KS/IHH should be further studied.

### 4.4. Genotype–Phenotype Correlations

KS patients displayed variable reproductive and non-reproductive phenotypes. Multiple phenotype representations of KS pose significant difficulties in establishing genotype–phenotype correlations.

The detection rate of P/LP variants of selected CPHD/IHH genes was higher in patients with severe non-reproductive phenotypes. Anosmia and additional congenital malformations appear to increase the chance of detecting a casual mutation, especially in GnRH neuron development and migration genes. P/LP variants were detected in patients presenting with anosmia/hyposmia in genes previously reported for nIHH (*GNRHR*) or SOD/CPHD (*LHX4*, *SOX3*). The coexistence of congenital anosmia (whose incidence is < 1/1,000,000) or another cause of smell loss and IHH cannot be ruled out [53,54]. Perhaps a specific sort of defect in *GNRHR*, *LHX4*, or *SOX3* can result in a complete clinical picture of Kallmann syndrome with smell disturbances. Further studies are required to explore this field.

A reversal of hypogonadism was seen in three of the studied patients (6.5%), slightly less often than expected (10–20%) according to the literature [14,55]. All of the subjects had a P/LP variant in *CHD7*, in two cases accompanied by P/LP variants in *FGFR1* (FGFR1: p.R285W) (unrelated patients 9 and 10). In the third case, a compound defect in *GNRHR* was associated with a mutation in *CHD7* (proband 25). Goncalves et al. described (similar to proband 4) a case of a male patient presenting with partial puberty and nIHH with a reversal in follow-up, diagnosed with a trigenic mutation (*FGFR1*: c.12G>T; *CHD7*: c.3245C>T; *PROKR2*: c.802C>T). Latinen et al., observed reversal in two patients with the same *GNRHR* mutation (p.R262Q), found also in the current study, which was accompanied by another *GNRHR* mutation (p.R139H or p. 309delF) and mutations in CHD7 (p.Q51X) or FGFR1 (c.91+2T>A) [56]. Patients with IHH/KS and defects in *CHD7* should be significantly monitored for a reversal of hypogonadism. According to the literature, the reversal of IHH is linked with mutations in *ANOS1*, *CHD7*, *FGFR1*, *FGF8*, *GNRHR*, *HS6ST1*, *KISS1*, *KISS1R*, *NSMF*, *PROK2*, *PROKR2*, *TAC3*, and *TACR3*, and such a minimum panel of 13 genes is recommended in diagnostics of reversal of IHH/KS [24,56,57,58].

The current study’s 13% rate of ao-IHH was comparable with the previously reported 10% incidence of ao-IHH in other IHH/KS study groups [12,59]. Subjects with ao-IHH seem to be more often hyposmic than anosmic, without additional congenital malformations or history of cryptorchidism. The heterogeneity of variants identified in ao-KS patients does not allow for the binding of this form of hypogonadism with a particular genetic defect in the examined population. Ao-IHH in the literature is linked with pathogenic changes in *CHD7*, *FGFR1*, *FGF8*, *FGF17*, *GNRHR*, *GNRH1*, *HS6ST1*, *NROB1*, *NSMF*, *PROK2*, *PROKR2*, and *WDR11* [14,25,34]. This is the first observation of ao-IHH in a hyposmic patient with a hemizygous pathogenic truncating variant (*SOX3*: p.R155Afs*26). The significance of *SOX3* mutations in the clinical picture of KS/IHH should be studied further.

The molecular diagnostics of KS are today effective and achievable. Costa-Barbosa et al. proposed targeting genetic diagnostics in KS regarding criteria relying on clinical phenotypes [15]. Detailed clinical evaluation of patients and searching for features of concomitant congenital malformations, differentiation of ao-IHH, and observation for reversal forms, are indispensable. Prioritizing genetic testing in KS patients is still challenging regarding the broad heterogeneity of detected defects, as also confirmed in the current study. Interestingly, patients with limb malformation presented mutations in *ANOS1*, *FGFR1*, *FGF8*, or *HESX1*, which is contrary to previous observations [15,60,61]. The presence of limb anomalies and any other major or additional CHARGE features enables the targeting of molecular diagnostics and testing for the *CHD7* gene mutation as the first choice. Limb anomalies are often described as part of the clinical picture of *CHD7* mutation and CHARGE syndrome [62]. In patients with synkinesia, searching for mutations in *ANOS1*, *FGFR1*/*FGF8*, and *PROK2/PROKR2* should be advised [4,15,60]. However, the presence of mirror movements did not exclude oligogenic mutations in *GNRHR* and *WDR11* (proband 11: *GNRHR*: p.Q106R and *GNRHR* p.Ser151 = and *WDR11*: p.I716V). Renal abnormalities are linked to KS and mutations in *ANOS1*, *CHD7*, *FGFR1*, and *FGF8* [4,34,63]. P/LP variants were also reported in *ANOS1*, *CHD7*, and *FGFR1*, as well as *GNRHR* in patients with renal phenotypes. In case of a lack of additional congenital malformation, panel-based NGS containing at least 15 of the most commonly altered genes (*ANOS1*, *CHD7*, *FGFR1*, *FGF8*, *GNRHR*, *GNRH1*, *HS6ST1*, *KISS1*, *KISS1R*, *NSMF*, *PROK2*, *PROKR2*, *TAC3*, *TACR3*, and *WDR11*) should be used in routine KS diagnostics.

### 4.5. Oligogenicity

Oligogenic, clinically relevant variants (P/LP) were seen in 11% of studied patients. The achieved diagnostic rate of oligogenicity was comparable to other IHH studies. However, the oligogenicity levels in KS/IHH may differ in various populations. Nair et al. described a 1.5% incidence of oligogenicity in Indians with IHH from Asia [11]. Sykiotis et al. used a panel of eight genes (*FGFR1*, *ANOS1*, *PROKR2*, *GNRHR*, *FGF8*, *KISS1R*, and *PROK2*) and found digenic mutations in 2% of all studied patients, and 11% of patients with a previously reported monogenic mutation [13]. Quayanor et al. found that the prevalence of digenic mutations detected with an NGS panel of 13 genes (*ANOS1*, *GNRHR*, *FGFR1*, *KISS1R*, *TAC3*, *TACR3*, *FGF8*, *PROKR2*, *PROK2*, *CHD7*, *NSMF*, *GNRH1*, and WDR11) *was at the level of* 12% [64]. As expected, digenic mutations are more frequent (11–16%) than trigenic ones (about 2% of patients) [13,14,31,58,64,65,66]. Currently, oligogenicity in IHH is considered to affect up to 10–20% of patients [14].

The set of 25 genes—i.e., *ANOS1*, *CHD7*, *DCC*, *DUSP6*, *FGFR1*, *FGF8*, *FGF17*, *FLRT3*, *GNRHR*, *HS6ST1*, *IL17RD*, *KISS1R*, *NSMF*, *NTN1*, *OL14RD*, *PNPL46*, *PROK2*, *PROKR2*, *SEMA3A*, *SEMA7A*, *SRA1*, *SPRY*, *TAC3*, *TACR3*, and *WDR11* have been described as inherited in the oligogenic IHH model. [25,67,68,69]. As far as is known, this is the first report evidencing digenic mutations in patients with KS and P/LP variants in PROKR2 and GLI2 (PROKR2: p.R85H and GLI2: p.A185S) genes (proband 30 with anosmia, absence of puberty, and micropenis).

The usage of tests employing analysis of a broad targeted panel of genes significantly raised the probabilityy of detection for clinically relevant changes in CPHD/IHH genes. Considering variants of all classes of pathogenicity, the prevalence of oligogenic variants was up to the level of 32.6%. The ACMG’s classification scheme is primarily intended to classify variants in Mendelian diseases’ genes, and was developed for the evaluation of variants in monogenic disorders [70].

For this reason, variants in all ACMG classes in IHH/CPHD have been reported in the current study. VUS alterations represent an unclear portion of defects, but considering their low frequency and in silico predictions, they could impair protein function, and are hypothesized to contribute to an oligogenic etiology and the development of the disease phenotype [71,72]. There are a growing number of studies, including VUS variants, investigating the possibility of providing a molecular diagnosis in case of reinterpretation of variants [73]. Future studies are needed in order to determine the pathogenicity of VUS variants to IHH/KS etiology.

The impact of the coexistence of different variants in a few genes on phenotype is still difficult to predict [13,14,16]. Two different genetic defects may have a synergistic effect [74]. In familial IHH cases, mutations in different loci give rise to slightly different phenotypes [65]. Synergistic heterozygosity, assuming the existence of several partial protein defects in at least one signaling pathway, has been thoroughly studied in cardiac and metabolic diseases and cancers [75,76,77]. Synergistic heterozygosity models in KS/IHH have been proposed for the mutations in *NSMF* and *ANOS1; NSMF* and *TACR3*; *FGF8*, *FGFR1*, and *ANOS1* [66,78]. A modifying, synergistic effect cannot be excluded regarding the coexistence of the P/LP, VUS, and perhaps even B/LB variants in critical genes essential for neuronal migration, the development of the pituitary gland, or signal transduction. The incidence of detected oligogenicity of KS in various studies relies significantly on the applied molecular method, assuming the number of analyzed genes, the variant classification criteria applied and, finally, the size of the examined cohort. There is a need for further research on KS/IHH using a comprehensive NGS approach with a wide panel of genes, whole-exome sequencing (WES), or even attempting patient whole-genome sequencing (WGS) if previous strategies fail to find a causative defect, in order to precisely define phenotypes [79].

The possible outcome of accumulated variants, principally P/LP and VUS, in more than one IHH/CPHD gene in the same patient should be continually studied. The significance of synergistic heterozygosity in IHH/KS pathogenesis represents a great challenge for further research.

## 5. Conclusions

In conclusion, this study has identified 20 new P/LP mutations in *ANOS1*, *CHD7*, *FGFR1*, *GLI2*, *GNRHR*, *SOX3*, and *WDR11*. The molecular basis of KS has been established for 43.5% of all studied patients. KS patients originating from Poland displayed variable reproductive and non-reproductive phenotypes, not always corresponding with previous data on phenotype–genotype correlation. The use of an NGS strategy employing a comprehensive panel of genes increases the chances of detecting monogenetic and oligogenic defects in KS patients presenting typical clinical characteristics. Mutations in genes responsible for GnRH neuron migration and development constitute the most frequent defects in studied subjects. The absence of recurrent alterations confirms the high heterogeneity of mutations, as evidenced in other studies. The relatively high incidence of oligogenicity in KS represents a diagnostic and interpretational challenge. The impact of oligogenicity and the relatively high VUS incidence on KS phenotype and disease course requires further observation. The role of CPHD genes, especially *LHX4* and *SOX3*, in KS development should be also further studied.

## Figures and Tables

**Table 1:** KS patients with genetic defects in primary GnRH neuron development/migration.

Patient No.	Stage	Variant HGVS	MAF	ACMG Classification	Anosmia/Hyposmia	Cryptorchidism	Associated Defects
**1**	**GnRH neuron migration**	ANOS1 p.R631* (c.1891C>T)	**NR**	**Pathogenic**	Anosmia	Bilateral	Bimanual synkinesis; left kidney agenesis
**2**	**GnRH neuron migration**	ANOS1 p.R631* (c.1891C>T)	**NR**	**Pathogenic**	Anosmia	Right testis	Bimanual synkinesis; right kidney agenesis
**3**	**GnRH neuron development**	FGFR1 p.W99* (c.297G>A)	**NR**	**Pathogenic**	Anosmia	Bilateral	Bilateral duplex pelvicalyceal system
**4**	**GnRH neuron development**	**FGFR1 p.V135I (c.403G>A)**	**0.00042**	**Likely pathogenic**	Hyposmia	No	NA
**5**	**GnRH neuron development**	**FGFR1 p. R281W (c.841C>T)**	**NR**	**Pathogenic**	Anosmia	Bilateral	Cleft lip and palate
	Pituitary dev. and signalling	GLI2 p.D1520N (c.4558G>A)	0.00935	Benign			
**6**	**GnRH neuron development**	**FGFR1 p.K649R (c.1946A>G)**	**NR**	**Likely pathogenic**	Hyposmia	Bilateral	NA
**7**	**GnRH neuron migration**	CHD7 p.V567Afs*8 (c.1699_1700insC)	**NR**	**Pathogenic**	Hyposmia	Bilateral	Double pyelum in the left kidney; myopia (8 dptr)
	GnRH neuron migration	CHD7 p.E1478= (c.4434A>G)	NR	Likely benign			
**8**	**GnRH neuron migration**	**CHD7 p.K850Q (c.2548A>C)**	**NR**	**Likely pathogenic**	Anosmia	No	Dental agenesis (lateral jaw’s incisors)
	**Pituitary dev. and signalling**	**LHX4 p.G305W (c.913G>T)**	**0.00000796**	**Likely pathogenic**			
**9**	**GnRH neuron migration**	**CHD7 p.N1030H (c.3088A>C)**	**NR**	**Pathogenic**	Anosmia	No	Sandal gap deformity
	**GnRH neuron development**	**FGFR1 p.R285W (c.853C>T)**	**NR**	**Likely pathogenic**			
**10**	**GnRH neuron migration**	**CHD7 p.N1030H (c.3088A>C)**	**NR**	**Pathogenic**	Anosmia	No	NA
	**GnRH neuron development**	**FGFR1 p.R285W (c.853C>T)**	**NR**	**Likely pathogenic**			
**11**	**GnRH neuron migration**	**CHD7 p.N1030H (c.3088A>C)**	**NR**	**Pathogenic**	Anosmia	No	Left kidney agenesis; splenomegaly
	GnRH neuron development	HS6ST1 p.K67* (c.199A>T)	0.00624	Benign			
	Pituitary dev. and signalling	LHX4 p.D128= (c.384C>T)	0.00864	Benign			
**12**	**GnRH neuron migration**	**CHD7 p.E1195A (c.3584A>C)**	**NR**	**Likely pathogenic**	Hyposmia	No	Syndactyly of the toes
**13**	**GnRH neuron migration**	CHD7 p.D2838Tfs*51 (c.8512delG)	**NR**	**Pathogenic**	Hyposmia	Right testis	Choanal atresia; clinodactyly; spina bifida (L5)
	Pituitary dev. and signalling	LHX4 p.D128= (c.384C>T)	0.00864	Benign			
14	GnRH neuron migration	CHD7 p.R947Q (c.2840G>A)	0.0000763	Uncertain significance	Anosmia	Bilateral	Micropenis; pre-auricular fistula; discoloration of the hair on the temple; brachydactyly
15	GnRH neuron migration	CHD7 p.M340V (c.1018A>G)	0.00462	Benign	Hyposmia	No	NA
16	GnRH neuron development	WDR11 p.M769V (c.2305A>G)	0.000565	Uncertain significance	Anosmia	Right testis	NA
	GnRH neuron migration	PROKR2 p.R268C (c.802C>T)	0.00391	Benign			
17	GnRH neuron development	FGF8 intronic (c.445-62G>T)	0.0000957	Uncertain significance	Anosmia	Migrating testis	NA
18	GnRH neuron development	FGF8 intronic (c.445-62G>T)	0.0000957	Uncertain significance	Anosmia	No	Intellectual disability
19	GnRH neuron development	FGF8 p.P26L (c.77C>T)	0.00115	Uncertain significance	Hyposmia	No	Bimanual synkinesis
20	GnRH neuron development	HS6ST1 p.R249S (c.745C>A)	0.0074	Uncertain significance	Anosmia	Right testis	NA
	GnRH neuron development	HS6ST1 p.D87E (c.261C>A)	0.01’	Uncertain significance			
21	GnRH neuron development	HS6ST1 p.R249S (c.745C>A)	0.0074	Uncertain significance	Anosmia	Right testis	Micropenis; syndactyly of toes
	GnRH neuron development	HS6ST1 p.D87E (c.261C>A)	0.01’	Uncertain significance			
**22**	**GnRH neuron development**	**WDR11 p.I716V (c.2146A>G)**	**NR**	**Likely pathogenic**	Anosmia	Right testis	Bimanual synkinesis
	**Pituitary dev. and signalling**	**GNRHR p.Q106R (c.317A>G)**	**0.00284**	**Likely pathogenic**			
	Pituitary dev. and signalling	GNRHR p.Ser151 = (c.453C>T)	0.0755	Uncertain significance			
23	GnRH neuron development	WDR11 p.M769V (c.2305A>G)	0.000565	Uncertain significance	Anosmia	No	NA

MAF: minor allele frequency; *: STOP codon; NR: not reported; ND: no data (neither results from the past nor information from the patient); NA: no abnormalities; variant frequency unclear because it falls within a segmental duplication region with inbreeding coefficient suspicion according to GnomAD; pathogenic/likely pathogenic variants are in bold.

**Table 2:** KS patients with genetic defects in hypothalamic/pituitary development and signaling.

Patient No.	Stage	Variant HGVS	MAF	ACMG Classification	Anosmia/Hyposmia	Cryptorchidism	Associated Defects
**24**	**Pituitary dev. and signalling**	GNRHR p.C114* (c342C>A)	**NR**	**Pathogenic**	Hyposmia	No	NA
	**Pituitary dev. and signalling**	**GNRHR p.R262Q (c.785G>A)**	**0.00179**	**Likely pathogenic**			
	Pituitary dev. and signalling	PCSK1 intronic (c.544-43T>G)	NR	Uncertain significance			
**25**	**Pituitary dev. and signalling**	**GNRHR p.R139H (c.416G>A)**	**0.000144**	**Pathogenic**	Hyposmia	No	NA
	**Pituitary dev. and signalling**	**GNRHR p.N10_Q11delinsKK (c.30_31delinsAA)**	**NR**	**Likely pathogenic**			
	GnRH neuron migration	CHD7 p.K683_T684insAK (c.2053_2058dupGCAAAA)	0.00623	Uncertain significance			
**26**	**Pituitary dev. and signalling**	**GNRHR p.P146S (c.436C>T)**	**0.00127**	**Pathogenic**	Anosmia	ND	Incomplete rotation of the right kidney
**27**	**Hypothalamic signalling**	GNRH1 p.C21Lfs*23 (c.60_61insC)	**0.00000401**	**Pathogenic**	Anosmia	ND	NA
	GnRH neuron development	WDR11 p.P475= (c.1425G>A)	0.00275	Benign			
28	Hypothalamic signalling	GNRH1 p.E47D (c.141G>C)	0.00153	Uncertain significance	Anosmia	No	Micropenis
	Hypothalamic signalling	GNRH1 p.F65= (c.183C>T)	0.00524	Uncertain significance			
29	Hypothalamic signalling	GNRH1 p.F65= (c.183C>T)	0.00524	Uncertain significance	Hyposmia	ND	NA
**30**	**Pituitary dev. and signalling**	**GLI2 p.G185C (c.553G>T)**	**NR**	**Likely pathogenic**	Anosmia	No	Micropenis
	**GnRH neuron migration**	**PROKR2 p.R85H (c.254G>A)**	**0.000712**	**Likely pathogenic**			
31	Pituitary dev. and signalling	GLI2 p.L1488F (c.4464G>T)	0.0000676	Uncertain significance	Hyposmia	No	NA
	Pituitary dev. and signalling	PITX2 p.T38= (c.114G>T)	0.000032	Likely benign			
32	Pituitary dev. and signalling	GLI2 p.G1006= (c.3018C>T)	0.00429	Benign	Anosmia	ND	Strabismus; oligodontia; ptosis; VSD
33	Pituitary dev. and signalling	GLI2 p.G1006= (c.3018C>T)	0.00429	Benign	Anosmia	ND	Strabismus; oligodontia; ptosis; VSD
**34**	**Pituitary dev. and signalling**	SOX3 p.R155Afs*26 (c.462_462delG)	**NR**	**Pathogenic**	Hyposmia	No	NA
35	Pituitary dev. and signalling	POLR3B p.T682A (c.2044A>G)	NR	Uncertain significance	Anosmia	No	NA
36	Pituitary dev. and signalling	NR0B1 p.S148N (c.443G>A)	NR	Uncertain significance	Anosmia	No	Abdominal hernia
	GnRH neuron migration	PROKR2 p.S130= (c.390C>T)	0.000529	Uncertain significance			
37	Pituitary dev. and signalling	LRRIQ3 p.R227C (c.679C>T)	0.000748	Uncertain significance	Hyposmia	No	NA
38	Pituitary dev. and signalling	LHX4 p.D128= (c.384C>T)	0.00864	Benign	Anosmia	Bilateral	NA
39	Pituitary dev. and signalling	LHX4 p.D128= (c.384C>T)	0.00864	Benign	Anosmia	No	NA
	GnRH neuron migration	CHD7p.S103T (c.307T>A)	0.0123	Benign			
40	Pituitary dev. and signalling	LHX3 p.Q41= (c.123G>A)	0.0111	Benign	Anosmia	Bilateral	NA

MAF: minor allele frequency; *: STOP codon; NR: not reported; ND: no data (neither results from the past nor information from the patient); NA: no abnormalities; variant frequency unclear because it falls within a segmental duplication region with inbreeding coefficient suspicion according to GnomAD; pathogenic/likely pathogenic variants are in bold.

## Data Availability

The data presented in this study are available on request from the corresponding author.

## Supplementary Materials

The following are available online at https://www.mdpi.com/article/10.3390/genes12060868/s1, Table S1: Variants Information.

## References

[1] Bianco S.D.C., Kaiser U.B. (2009). The genetic and molecular basis of idiopathic hypogonadotropic hypogonadism. Nat. Rev. Endocrinol..

[2] Schwanzel-Fukuda M., Pfaff D.W. (1989). Origin of luteinizing hormone-releasing hormone neurons. Nat. Cell Biol..

[3] Wray S. (2002). Development of gonadotropin-releasing hormone-1 neurons. Front. Neuroendocr..

[4] Tickotsky N., Moskovitz M. (2014). Renal Agenesis in Kallmann Syndrome: A Network Approach. Ann. Hum. Genet..

[5] Quinton R., Duke V.M., Robertson A., Kirk J.M.W., Matfin G., De Zoysa P.A., Azcona C., MacColl G.S., Jacobs H.S., Conway G.S. (2001). Idiopathic gonadotrophin deficiency: Genetic questions addressed through phenotypic characterization. Clin. Endocrinol..

[6] Della Valle E., Vezzani S., Rochira V., Granata A.R.M., Madeo B., Genovese E., Pignatti E., Marino M., Carani C., Simoni M. (2013). Prevalence of Olfactory and Other Developmental Anomalies in Patients with Central Hypogonadotropic Hypogonadism. Front. Endocrinol..

[7] Kim H.-G., Kurth I., Lan F., Meliciani I., Wenzel W., Eom S.H., Kang G.B., Rosenberger G., Tekin M., Ozata M. (2008). Mutations in CHD7, Encoding a Chromatin-Remodeling Protein, Cause Idiopathic Hypogonadotropic Hypogonadism and Kallmann Syndrome. Am. J. Hum. Genet..

[8] Pinto G., Abadie V., Mesnage R., Blustajn J., Cabrol S., Amiel J., Hertz-Pannier L., Bertrand A.M., Lyonnet S., Rappaport R. (2005). Charge Syndrome Includes Hypogonadotropic Hypogonadism and Abnormal Olfactory Bulb Development. J. Clin. Endocrinol. Metab..

[9] Cho H.-J., Shan Y., Whittington N.C., Wray S. (2019). Nasal Placode Development, GnRH Neuronal Migration and Kallmann Syndrome. Front. Cell Dev. Biol..

[10] Laitinen E.-M., Vaaralahti K., Tommiska J., Eklund E., Tervaniemi M., Valanne L., Raivio T. (2011). Incidence, Phenotypic Features and Molecular Genetics of Kallmann Syndrome in Finland. Orphanet J. Rare Dis..

[11] Nair S., Jadhav S., Lila A., Jagtap V., Bukan A., Pandit R., Ekbote A., Dharmalingam M., Kumar P., Kalra P. (2016). Spectrum of phenotype and genotype of congenital isolated hypogonadotropic hypogonadism in Asian Indians. Clin. Endocrinol..

[12] Bonomi M., Vezzoli V., Krausz C., Guizzardi F., Vezzani S., Simoni M., Bassi I., Duminuco P., di Iorgi N., Giavoli C. (2018). Characteristics of a Nationwide Cohort of Patients Presenting with Isolated Hy-pogonadotropic Hypogonadism (Ihh). Eur. J. Endocrinol..

[13] Sykiotis G.P., Plummer L., Hughes V.A., Au M., Durrani S., Nayak-Young S., Dwyer A.A., Quinton R., Hall J.E., Gusella J.F. (2010). Oligogenic Basis of Isolated Gonadotropin-Releasing Hormone Deficiency. Proc. Natl. Acad. Sci. USA.

[14] Topaloğlu A.K. (2018). Update on the Genetics of Idiopathic Hypogonadotropic Hypogonadism. J. Clin. Res. Pediatr. Endocrinol..

[15] Costa-Barbosa F.A., Balasubramanian R., Keefe K.W., Shaw N., Al Tassan N., Plummer L., Dwyer A.A., Buck C.L., Choi J.-H., Seminara S.B. (2013). Prioritizing Genetic Testing in Patients with Kallmann Syndrome Using Clinical Phenotypes. J. Clin. Endocrinol. Metab..

[16] Maione L., Dwyer A.A., Francou B., Guiochon-Mantel A., Binart N., Bouligand J., Young J. (2018). Genetics in Endocrinology: Genetic counseling for congenital hypogonadotropic hypogonadism and Kallmann syndrome: New challenges in the era of oligogenism and next-generation sequencing. Eur. J. Endocrinol..

[17] Rojek A., Obara-Moszynska M., Malecka E., Slomko-Jozwiak M., Niedziela M. (2013). NR0B1 (DAX1) mutations in patients affected by congenital adrenal hypoplasia with growth hormone deficiency as a new finding. J. Appl. Genet..

[18] Dzaman K., Zborowska-Piskadlo K., Pietniczka-Zaleska M., Kantor I. (2017). Kallmann Syndrome in Pediatric Otorhinolar-yngology Practice—Case Report and Literature Review. Int. J. Pediatr. Otorhinolaryngol..

[19] Placzkiewicz E., Baldys-Waligorska A. (2003). Kallmann’s Syndrome: Skeletal and Psychological Aspects of Late Diagnosis. Ann. Endocrinol..

[20] Noczynska A., Wasikowa R. (2004). Kallmann’s Syndrome in Families. Endokrynol. Diabetol. Chor. Przemiany Mater. Wieku Rozw..

[21] Pierzchlewska M.M., Robaczyk M.G., Vogel I. (2015). Induction of puberty with human chorionic gonadotropin (hCG) followed by reversal of hypogonadotropic hypogonadism in Kallmann syndrome. Endokrynol. Polska.

[22] Gach A., Pinkier I., Sałacińska K., Szarras-Czapnik M., Salachna D., Kucińska A., Rybak-Krzyszkowska M., Sakowicz A. (2020). Identification of gene variants in a cohort of hypogonadotropic hypogonadism: Diagnostic utility of custom NGS panel and WES in unravelling genetic complexity of the disease. Mol. Cell. Endocrinol..

[23] Gach A., Pinkier I., Szarras-Czapnik M., Sakowicz A., Jakubowski L. (2020). Expanding the mutational spectrum of monogenic hypogonadotropic hypogonadism: Novel mutations in ANOS1 and FGFR1 genes. Reprod. Biol. Endocrinol..

[24] Mao J.F., Xu H.L., Duan J., Chen R.R., Li L., Li B., Nie M., Min L., Zhang H.B., Wu X.Y. (2015). Reversal of Idiopathic Hy-pogonadotropic Hypogonadism: A Cohort Study in Chinese Patients. Asian J. Androl..

[25] Boehm U., Bouloux P.M., Dattani M.T., de Roux N., Dode C., Dunkel L., Dwyer A.A., Giacobini P., Hardelin J.P., Juul A. (2015). Expert Consensus Document: Eu-ropean Consensus Statement on Congenital Hypogonadotropic Hypogonadism—Pathogenesis, Diagnosis and Treatment. Nat. Rev. Endocrinol..

[26] Elsberg C.A., Levy I., Brewer E.D., Merrill M.H., Lacaillade C.W., Broeck C.T. (1936). A new method for testing the sense of SMELL and for the establishment of olfactory values of odorous substances. Science.

[27] Pruszewicz A. (1965). Apropos of Gustatory and Olfactory Tests. Otolaryngol. Polska.

[28] Kopanos C., Tsiolkas V., Kouris A., Chapple C.E., Aguilera M.A., Meyer R., Massouras A. (2019). VarSome: The human genomic variant search engine. Bioinformatics.

[29] Li Q., Wang K. (2017). InterVar: Clinical Interpretation of Genetic Variants by the 2015 ACMG-AMP Guidelines. Am. J. Hum. Genet..

[30] Yang Z., Lasker K., Schneidman-Duhovny D., Webb B., Huang C.C., Pettersen E.F., Goddard T.D., Meng E.C., Sali A., Ferrin T.E. (2012). UCSF Chimera, MODELLER, and IMP: An integrated modeling system. J. Struct. Biol..

[31] Quaynor S.D., Bosley M.E., Duckworth C.G., Porter K.R., Kim S.-H., Kim H.-G., Chorich L.P., Sullivan M.E., Choi J.-H., Cameron R.S. (2016). Targeted next generation sequencing approach identifies eighteen new candidate genes in normosmic hypogonadotropic hypogonadism and Kallmann syndrome. Mol. Cell. Endocrinol..

[32] Butz H., Nyírő G., Kurucz P.A., Likó I., Patócs A. (2021). Molecular genetic diagnostics of hypogonadotropic hypogonadism: From panel design towards result interpretation in clinical practice. Qual. Life Res..

[33] Cangiano B., Duminuco P., Vezzoli V., Guizzardi F., Chiodini I., Corona G., Maggi M., Persani L., Bonomi M. (2019). Evidence for a Common Genetic Origin of Classic and Milder Adult-Onset Forms of Isolated Hypogonadotropic Hypogonadism. J. Clin. Med..

[34] Balasubramanian R., Crowle W. (2007). Isolated Gonadotropin-Releasing Hormone (Gnrh) Deficiency.

[35] Xu C., Cassatella D., van der Sloot A.M., Quinton R., Hauschild M., de Geyter C., Fluck C., Feller K., Bartholdi D., Nemeth A. (2018). Evaluating Charge Syndrome in Congenital Hypogonadotropic Hypogonadism Patients Harboring Chd7 Variants. Genet. Med..

[36] Kim H.G., Layman L.C. (2011). The Role of Chd7 and the Newly Identified Wdr11 Gene in Patients with Idiopathic Hypogonadotropic Hypogonadism and Kallmann Syndrome. Mol. Cell Endocrinol..

[37] Turan I., Hutchins B.I., Hacihamdioglu B., Kotan L.D., Gurbuz F., Ulubay A., Mengen E., Yuksel B., Wray S., Topaloglu A.K. (2017). CCDC141 Mutations in Idiopathic Hypogonadotropic Hypogonadism. J. Clin. Endocrinol. Metab..

[38] Tusset C., Trarbach E.B., Silveira L.F., Beneduzzi D., Montenegro L., Latronico A.C. (2011). Clinical and Molecular Aspects of Congenital Isolated Hypogonadotropic Hypogonadism. Arq. Bras. Endocrinol. Metabol..

[39] Dode C., Teixeira L., Levilliers J., Fouveaut C., Bouchard P., Kottler M.L., Lespinasse J., Lienhardt-Roussie A., Mathieu M., Moerman A. (2006). Kallmann Syndrome: Mutations in the Genes Encoding Prokineticin-2 and Prokineticin Receptor-2. PLoS Genet..

[40] Monnier C., Dodé C., Fabre L., Teixeira L., Labesse G., Pin J.-P., Hardelin J.-P., Rondard P. (2008). PROKR2 missense mutations associated with Kallmann syndrome impair receptor signalling activity. Hum. Mol. Genet..

[41] Raivio T., Avbelj M., McCabe M.J., Romero C.J., Dwyer A.A., Tommiska J., Sykiotis G.P., Gregory L.C., Diaczok D., Tziaferi V. (2012). Genetic Overlap in Kallmann Syndrome, Combined Pituitary Hormone Deficiency, and Septo-Optic Dysplasia. J. Clin. Endocrinol. Metab..

[42] Vaaralahti K., Raivio T., Koivu R., Valanne L., Laitinen E.-M., Tommiska J. (2012). Genetic Overlap between Holoprosencephaly and Kallmann Syndrome. Mol. Syndr..

[43] Asakura Y., Muroya K., Hanakawa J., Sato T., Aida N., Narumi S., Hasegawa T., Adachi M. (2015). Combined Pituitary Hormone Deficiency with Unique Pituitary Dysplasia and Morning Glory Syndrome Related to a Heterozygous Prokr2 Mutation. Clin. Pediatr. Endocrinol..

[44] Correa F.A., Trarbach E.B., Tusset C., Latronico A.C., Montenegro L.R., Carvalho L.R., Franca M.M., Otto A.P., Costalonga E.F., Brito V.N. (2015). FGFR1 and PROKR2 rare variants found in patients with combined pituitary hormone deficiencies. Endocr. Connect..

[45] Kim J.H., Seo G.H., Kim G.-H., Huh J., Hwang I.T., Jang J.-H., Yoo H.-W., Choi J.-H. (2018). Targeted Gene Panel Sequencing for Molecular Diagnosis of Kallmann Syndrome and Normosmic Idiopathic Hypogonadotropic Hypogonadism. Exp. Clin. Endocrinol. Diabetes.

[46] Vaaralahti K., Tommiska J., Tillmann V., Liivak N., Känsäkoski J., Laitinen E.-M., Raivio T. (2014). De novo SOX10 nonsense mutation in a patient with Kallmann syndrome and hearing loss. Pediatr. Res..

[47] Pingault V., Bodereau V., Baral V., Marcos S., Watanabe Y., Chaoui A., Fouveaut C., Leroy C., Vérier-Mine O., Francannet C. (2013). Loss-of-Function Mutations in SOX10 Cause Kallmann Syndrome with Deafness. Am. J. Hum. Genet..

[48] Suzuki E., Izumi Y., Chiba Y., Horikawa R., Matsubara Y., Tanaka M., Ogata T., Fukami M., Naiki Y. (2015). Loss-of-Function SOX10 Mutation in a Patient with Kallmann Syndrome, Hearing Loss, and Iris Hypopigmentation. Horm. Res. Paediatr..

[49] Wang F., Zhao S., Xie Y., Yang W., Mo Z. (2018). De novo SOX10 Nonsense Mutation in a Patient with Kallmann Syndrome, Deafness, Iris Hypopigmentation, and Hyperthyroidism. Ann. Clin. Lab. Sci..

[50] Lrriq3. https://www.proteinatlas.org/ENSG00000162620-LRRIQ3/tissue.

[51] Reuter M.S., Tawamie H., Buchert R., Gebril O.H., Froukh T., Thiel C., Uebe S., Ekici A.B., Krumbiegel M., Zweier C. (2017). Diagnostic Yield and Novel Candidate Genes by Exome Sequencing in 152 Consanguineous Families with Neurodevelopmental Disorders. JAMA Psychiatry.

[52] Howard S.R., Guasti L., Ruiz-Babot G., Mancini A., David A., Storr H.L., Metherell L.A., Sternberg M.J., Cabrera C.P., Warren H.R. (2016). Igsf10 Mutations Dysregulate Gonadotropin-Releasing Hormone Neuronal Migration Resulting in Delayed Puberty. EMBO Mol. Med..

[53] Karstensen H., Tommerup N. (2011). Isolated and syndromic forms of congenital anosmia. Clin. Genet..

[54] Dalton P., Mennella J.A., Cowart B.J., Maute C., Pribitkin E.A., Reilly J.S. (2009). Evaluating the Prevalence of Olfactory Dysfunction in a Pediatric Population. Ann. N. Y. Acad. Sci..

[55] Raivio T., Falardeau J., Dwyer A., Quinton R., Hayes F.J., Hughes V.A., Cole L.W., Pearce S.H., Lee H., Boepple P. (2007). Reversal of Idiopathic Hypogonadotropic Hypogonadism. N. Engl. J. Med..

[56] Laitinen E.-M., Tommiska J., Sane T., Vaaralahti K., Toppari J., Raivio T. (2012). Reversible Congenital Hypogonadotropic Hypogonadism in Patients with CHD7, FGFR1 or GNRHR Mutations. PLoS ONE.

[57] Bouligand J., Ghervan C., Tello J.A., Brailly-Tabard S., Salenave S., Chanson P., Lombès M., Millar R.P., Guiochon-Mantel A., Young J. (2009). Isolated Familial Hypogonadotropic Hypogonadism and aGNRH1Mutation. N. Engl. J. Med..

[58] Cole L.W., Sidis Y., Zhang C., Quinton R., Plummer L., Pignatelli D., Hughes V.A., Dwyer A.A., Raivio T., Hayes F.J. (2008). Mutations in Prokineticin 2andProkineticin receptor 2genes in Human Gonadotrophin-Releasing Hormone Deficiency: Molecular Genetics and Clinical Spectrum. J. Clin. Endocrinol. Metab..

[59] Nachtigall L.B., Boepple P.A., Pralong F.P., Crowley W.F. (1997). Adult-Onset Idiopathic Hypogonadotropic Hypogonadism—A Treatable Form of Male Infertility. N. Engl. J. Med..

[60] Knickmeyer R.C., Auyeung B., Davenport M.L. (2015). Assessing Prenatal and Neonatal Gonadal Steroid Exposure for Studies of Human Development: Methodological and Theoretical Challenges. Front. Endocrinol..

[61] Trarbach E.B., Abreu A.P., Silveira L.F., Garmes H.M., Baptista M.T., Teles M.G., Costa E.M., Mohammadi M., Pitteloud N., Mendonca B.B. (2010). Nonsense Mutations in Fgf8 Gene Causing Different Degrees of Human Gonadotropin-Releasing Deficiency. J. Clin. Endocrinol. Metab..

[62] Brock K.E., Mathiason M.A., Rooney B.L., Williams M.S. (2009). Quantitative analysis of limb anomalies in CHARGE syndrome: Correlation with diagnosis and characteristic CHARGE anomalies. Am. J. Med. Genet. Part A.

[63] Sanna-Cherchi S., Caridi G., Weng P.L., Scolari F., Perfumo F., Gharavi A.G., Ghiggeri G.M. (2007). Genetic Approaches to Human Renal Agenesis/Hypoplasia and Dysplasia. Pediatr. Nephrol..

[64] Quaynor S.D., Kim H.G., Cappello E.M., Williams T., Chorich L.P., Bick D.P., Sherins R.J., Layman L.C. (2011). The Prevalence of Digenic Mutations in Patients with Normosmic Hypogonadotropic Hypogonadism and Kallmann Syndrome. Fertil. Steril..

[65] Pitteloud N., Quinton R., Pearce S., Raivio T., Acierno J., Dwyer A., Plummer L., Hughes V., Seminara S., Cheng Y.-Z. (2007). Digenic mutations account for variable phenotypes in idiopathic hypogonadotropic hypogonadism. J. Clin. Investig..

[66] Xu N., Kim H.-G., Bhagavath B., Cho S.-G., Lee J.H., Ha K., Meliciani I., Wenzel W., Podolsky R.H., Chorich L.P. (2011). Nasal embryonic LHRH factor (NELF) mutations in patients with normosmic hypogonadotropic hypogonadism and Kallmann syndrome. Fertil. Steril..

[67] Miraoui H., Dwyer A.A., Sykiotis G.P., Plummer L., Chung W., Feng B., Beenken A., Clarke J., Pers T.H., Dworzynski P. (2013). Mutations in Fgf17, Il17rd, Dusp6, Spry4, and Flrt3 Are Identified in Individuals with Congenital Hypogonadotropic Hy-pogonadism. Am. J. Hum. Genet..

[68] Bouilly J., Messina A., Papadakis G., Cassatella D., Xu C., Acierno J.S., Tata B., Sykiotis G., Santini S., Sidis Y. (2018). Dcc/Ntn1 Complex Mutations in Patients with Congenital Hypogonadotropic Hypogonadism Impair Gnrh Neuron Development. Hum. Mol. Genet..

[69] Kotan L.D., Hutchins B.I., Ozkan Y., Demirel F., Stoner H., Cheng-Hathaway P., Esen I., Gurbuz F., Bicakci Y.K., Mengen E. (2014). Mutations in FEZF1 Cause Kallmann Syndrome. Am. J. Hum. Genet..

[70] Amendola L.M., Jarvik G.P., Leo M.C., McLaughlin H.M., Akkari Y., Amaral M.D., Berg J.S., Biswas S., Bowling K.M., Conlin L.K. (2016). Performance of ACMG-AMP Variant-Interpretation Guidelines among Nine Laboratories in the Clinical Sequencing Exploratory Research Consortium. Am. J. Hum. Genet..

[71] Szot J., Cuny H., Blue G.M., Humphreys D.T., Ip E., Harrison K., Sholler G.F., Giannoulatou E., Leo P., Duncan E.L. (2018). A Screening Approach to Identify Clinically Actionable Variants Causing Congenital Heart Disease in Exome Data. Circ. Genom. Precis. Med..

[72] Alosi D., Bisgaard M.L., Hemmingsen S.N., Krogh L.N., Mikkelsen H.B., Binderup M.L.M. (2017). Management of Gene Variants of Unknown Significance: Analysis Method and Risk Assessment of the Vhl Mutation P.P81s (C.241c>T). Curr. Genom..

[73] Vears D.F., Niemiec E., Howard H.C., Borry P. (2018). Analysis of VUS reporting, variant reinterpretation and recontact policies in clinical genomic sequencing consent forms. Eur. J. Hum. Genet..

[74] Pérez-Pérez J.M., Candela H., Micol J.L. (2009). Understanding synergy in genetic interactions. Trends Genet..

[75] Pulignani S., Vecoli C., Borghini A., Foffa I., Ait-Ali L., Andreassi M.G. (2018). Targeted Next-Generation Sequencing in Patients with Non-syndromic Congenital Heart Disease. Pediatr. Cardiol..

[76] Cervera-Acedo C., Coloma A., Huarte-Loza E., Sierra-Carpio M., Domínguez-Garrido E. (2017). Phenotype variability in a large Spanish family with Alport syndrome associated with novel mutations in COL4A3 gene. BMC Nephrol..

[77] Niku M., Pajari A.-M., Sarantaus L., Päivärinta E., Storvik M., Heiman-Lindh A., Suokas S., Nyström M., Mutanen M. (2017). Western diet enhances intestinal tumorigenesis in Min/+ mice, associating with mucosal metabolic and inflammatory stress and loss of Apc heterozygosity. J. Nutr. Biochem..

[78] González-Martínez D., Kim S.-H., Hu Y., Guimond S., Schofield J., Winyard P., Vannelli G.B., Turnbull J., Bouloux P.-M. (2004). Anosmin-1 Modulates Fibroblast Growth Factor Receptor 1 Signaling in Human Gonadotropin-Releasing Hormone Olfactory Neuroblasts through a Heparan Sulfate-Dependent Mechanism. J. Neurosci..

[79] Cassatella D., Howard S., Acierno J.S., Xu C., Papadakis G.E., Santoni F.A., Dwyer A.A., Santini S., Sykiotis G.P., Chambion C. (2018). Congenital hypogonadotropic hypogonadism and constitutional delay of growth and puberty have distinct genetic architectures. Eur. J. Endocrinol..

